# *Child and Adolescent Psychiatry and Mental Health* reviewer acknowledgement 2015

**DOI:** 10.1186/s13034-016-0094-7

**Published:** 2016-03-11

**Authors:** Jörg M. Fegert

**Affiliations:** Department of Child and Adolescent Psychiatry and Psychotherapy, University of Ulm, Steinhoevelstr. 5, 89075 Ulm, Germany

## Contributing reviewers

The editors of *Child and Adolescent Psychiatry and Mental Health* would like to thank all of our reviewers who have contributed to the journal in Volume 9 (2015).

**Kenneth J. Aitken**

United Kingdom

**Marc Allroggen**

Germany

**Claire Amstrong-Kelly**

Australia

**Anna-Karin Andershed**

Sweden

**Margaret Andover**

United States of America

**Phil Asherson**

United Kingdom

**Andrea Asnes**

United States

**Olayinka Atilola**

Nigeria

**Marc Atkins**

United States of America

**Imke Baetens**

Belgium

**Judit Balazs**

Hungary

**Paula Barrett**

Australia

**Lucas Battel**

Brazil

**Myron Belfer**

United States of America

**Isabel Boege**

Germany

**David Brent**

United States of America

**Konrad Bresin**

United States of America

**Katharine-Ann Buck**

United States

**Simona Bujoreanu**

United States of America

**Alison Calear**

Australia

**Gabrielle Campbell**

Australia

**Franco Cavallo**

Italy

**Henri Chabrol**

France

**Megan Chesin**

United States of America

**Chee Hon Chin**

Singapore

**Georgia Chronaki**

United Kingdom

**Joanna Ting Wai Chu**

New Zealand

**Laurence Claes**

Belgium

**Antonio Clavenna**

Italy

**Paula Cloutier**

Canada

**Ana Cubillo**

Switzerland

**Graccielle R Cunha**

Brazil

**Örjan Dahlström**

Sweden

**Naomi Davis**

United States of America

**William Daviss**

United States of America

**Michelle Dawson**

Canada

**Diego De Leo**

Australia

**Susanne De Rooij**

The Netherlands

**Wieke De Vente**

The Netherlands

**Julia Diehle**

United Kingdom

**Janean Dilworth-Bart**

United States

**Sarah Doucette**

Canada

**Stefan Ecks**

United Kingdom

**Sigmund Eldevik**

Norway

**Pia Enebrink**

Sweden

**Eyup Sabri Ercan**

Turkey

**Spencer Evans**

United States of America

**Michelle Evans-Chase**

United States of America

**Minne Fekkes**

The Netherlands

**Reiner Frank**

Germany

**Andrew Fugard**

United Kingdom

**Daniel Fung**

Singapore

**Penny Furness**

United Kingdom

**Joy Gabrielli**

United States of America

**Amarendra Gandhi**

Belgium

**Bernice Garnett**

United States of America

**Christiane Geyer**

Brazil

**Catherine Glenn**

United States of America

**Kirstin Goth**

Switzerland

**Laurence Greenhill**

United States of America

**Rebecca Groschwitz**

Germany

**Johannes Haerting**

Germany

**Charlotte Hall**

United Kingdom

**Ben Hannigan**

United Kingdom

**Rochelle Hanson**

United States of America

**Antonio Hardan**

United States

**Penelope Hasking**

Australia

**Christopher Hautmann**

Germany

**Daniel Hayes**

United Kingdom

**Nancy Heath**

Canada

**Craig Anne Heflinger**

United States of America

**Hartmut Heinrich**

Germany

**Nina Heinrichs**

Germany

**Sam Himelstein**

United States of America

**Maurício Hoffmann**

Brazil

**Jacqueline Horan**

United States of America

**Patricia Howlin**

United Kingdom

**Tom Hummer**

United States of America

**Jeffrey Hunt**

United States of America

**Hiroko Ichikawa**

Japan

**Nazish Imran**

Pakistan

**Daniza M Ivanovic**

Chile

**Anna-Karin Ivert**

Sweden

**Thomas Jans**

Germany

**Vanessa Jantzer**

Germany

**Abigail Jenkins**

United States of America

**Ann John**

United Kingdom

**Ulf Jonsson**

Sweden

**Andreas Jud**

Switzerland

**Jon Jureidini**

Australia

**Michael Kaess**

Germany

**Ferdinand Keller**

Germany

**Praveen Khairkar**

India

**Glenn Kiekens**

Belgium

**Christian Kieling**

Brazil

**Jennifer Kiing**

Singapore

**Ryan Kilmer**

United States of America

**Evan Kleiman**

United States of America

**E. David Klonsky**

Canada

**Michael Koelch**

Germany

**Liv Kosnes**

United Kingdom

**Hanna Kovshoff**

United Kingdom

**Solvejg Kristensen**

Denmark

**Anne Künster**

Germany

**Odilia M. Laceulle**

The Netherlands

**Thao Le**

United States of America

**Stephen P. Lewis**

Canada

**Sabine Loos**

Germany

**Laura López-Romero**

Spain

**Paul Lysaker**

United States

**Aristides Machado-Rodrigues**

Portugal

**Harriet Macmillan**

Canada

**Gisele Manfro**

Brazil

**Andres Martin**

United States of America

**Paul Maruff**

Australia

**Molly Meers**

United States of America

**Sunil Mehta**

United States

**Richard Meiser-Stedman**

United Kingdom

**Yukifumi Monden**

Japan

**Taís Moriyama**

Brazil

**Jennifer Muehlenkamp**

United States of America

**Ian Munt**

Singapore

**Stella Muthuri**

Canada

**Matthew Mychailyszyn**

United States of America

**Carl Myers**

United States

**Maaike H. Nauta**

The Netherlands

**Tamsin Newlove-Delgado**

United Kingdom

**Jared Ng**

Singapore

**Dennis Nitkowski**

Germany

**Chiara Nosarti**

United Kingdom

**Bridianne O’dea**

Australia

**Susanne Ohmann**

Austria

**Dennis Ougrin**

United Kingdom

**Susanna Payne**

United Kingdom

**Katherine Pears**

United States

**Friedemann Pfäfflin**

Germany

**Lue Ping Ong**

Singapore

**Paul L. Plener**

Germany

**Beth A. Pletcher**

United States

**Irina Elizabeth Poslawsky**

The Netherlands

**Michèle Preyde**

Canada

**Ravi P. Rajkumar**

India

**Alessandra Raudino**

Australia

**Reuben Robbins**

United States of America

**Cynthia Rogers**

United States

**Rita Rosner**

Germany

**Neal D. Ryan**

United States

**Christina Sakai**

United States of America

**Vedat Sar**

Turkey

**Ingo Schäfer**

Germany

**Michael Scheeringa**

United States of America

**Klaus Schmeck**

Switzerland

**Marc Schmid**

Switzerland

**Michael Schoenbaum**

United States of America

**Felipe Schuch**

Brazil

**Adina Seidenfeld**

United States of America

**Kaushik Shah**

United States of America

**Stephanie Shire**

United States of America

**Federico Sicca**

Italy

**Anna Sidor**

Germany

**Ellin Simon**

Belgium

**Johan Simons**

Belgium

**Michael Simons**

Germany

**April Smith**

United States of America

**Tristram Smith**

United States of America

**Anthony Spirito**

United States of America

**Christina Stadler**

Switzerland

**Jeremy Stewart**

United States of America

**Joana Straub**

Germany

**Mythily Subramaniam**

Singapore

**Michaela Swales**

United Kingdom

**James Swanson**

United States of America

**Toshinobu Takeda**

Japan

**Kanako Taku**

United States of America

**Lil Tonmyr**

Canada

**James Topitzes**

United States

**Brianna Turner**

Canada

**Alasdair Vance**

Australia

**Marianne Villabø**

Norway

**Benedetto Vitiello**

United States

**Sebastian Vollmer**

Germany

**Jan Wallander**

United States of America

**Zachary Warren**

United States of America

**Jason Washburn**

United States

**James Waxmonsky**

United States of America

**Chang Weining**

Singapore

**Susan White**

United States of America

**Janis Whitlock**

United States of America

**Mechthild Wolff**

Germany

**Alisa Woods**

United States of America

**Frances Wymbs**

United States of America

**Maria Zetterqvist**

Sweden

**Julie Zito**

United States of America

